# Supplementary material for: Diversity Waves in Collapse-Driven Population Dynamics
Source: PLoS Comput Biol. 2015 Sep 14;11(9):e1004440. doi: 10.1371/journal.pcbi.1004440 (PMC4569562; doi:10.1371/journal.pcbi.1004440)
Supplement: S1 Text — (PDF) [file pcbi.1004440.s001.pdf]

S1 Text

Fokker-Planck equation for the basic model

Let's consider a version of our model with a very low value of γ to ensure the complete separation between the upper and lower peaks. The multiplicative dynamics of surviving populations in the upper peak is described by the following elementary step:

$$P_i(t) = \frac{P_i(t-1)}{1 - P_{j(t)}(t)}$$

. Here $P_j(t)(t)$ is the population at time step t of the species $j(t)$ that went extinct at this time step. It can be also easily integrated for all times since the beginning of the current wave at time step 1:

$$P_i(t) = \frac{P_i(1)}{1 - \sum_{t'=1}^{t} P_{j(t')}(1)} \quad . \tag{S1}$$

Indeed, $\sum_{t'=1}^{t} P_{j(t')}(1)$ is the total initial (at time step 1) populations of all species that went extinct by the time step t. Hence $1 - \sum_{t'=1}^{t} P_{j(t')}(1)$ - is the total initial population of all surviving species used to normalize their initial populations to give their populations at the time step t (population ratios of surviving species are preserved in our basic model). Taking the logarithm of both sides of Eq. S1 and approximating $-\log(1 - \sum_{t'=1}^{t} P_{j(t')}(1)) \simeq \sum_{t'=1}^{t} P_{j(t')}(1)$, which holds as long as the system is still far away from the end of the wave ($\sum_{t'=1}^{t} P_{j(t')}(1) \ll 1$, one gets:

$$\log P_i(t) = \log P_i(1) + \sum_{t'=1}^{t} P_{j(t')}(1) \quad . \tag{S2}$$

The stochastic dynamics within a single wave can thus be described by the following equation:

$$\frac{d \log(P_i(t))}{dt} = P_{j(t)}(1) \quad . \tag{S3}$$

The exponent τ of the population size distribution in our model is determined by the balance between the noisy multiplicative population dynamics and the exponential loss of surviving species due to collapses.

It can be approximated by the following Fokker-Plank-like equation:

$$\frac{\partial \pi}{\partial t} = -\rho\pi - \mu\frac{\partial \pi}{\partial \log P} + \sigma\frac{\partial^2 \pi}{(\partial \log P)^2} + \text{episodic source terms} \qquad \text{(S4)}$$

Here $\pi(\log P, t)$ is the time-dependent population abundance distribution in the upper peak, ρ - the loss term due to population collapses, μ - the logarithmic drift velocity and σ - is the logarithmic dispersion, which is totally absent in the simplified model where all populations start at the same size.

The Eqs.S2-S3 allow us to derive the parameters of the Fokker-Plank equation in terms of the distribution of population sizes $P_i(1)$ at the start of the wave. Indeed, early in the wave one has $\rho = 1/N$, $\mu = \langle P_i(1)\rangle_i = 1/N$ and $\sigma = \langle P_i(1)^2\rangle_i - \langle P_i(1)\rangle_i^2 = (1/N)\cdot(1/D(1) - 1/N)$. Note an unusual connection between the population diversity at the start of a wave $D(1)$, and the diffusion coefficient σ in the Fokker-Plank equation.

The stationary solution for the time-aggregate distribution $\int \pi(\log P, t)dt$ has an exponential tail $\exp(-(\tau - 1)\log P)$. It corresponds to the power law tail of the species population distribution $\propto P^{-\tau}$. The exponent τ is defined by one of the two solutions to the quadratic equation

$$0 = -\frac{1}{N} + \frac{1}{N}(\tau - 1) + \sigma(\tau - 1)^2 \quad . \qquad \text{(S5)}$$

In the version of the model, where all populations at the start of the wave are equal to each other, sizes of surviving populations increase deterministically as $\exp(t/N)/N$ (see main text for the derivation) and thus have zero dispersion: $\sigma = 0$. Hence in this simplified version the exponent $\tau = 1 + \rho/\mu = 2$ is determined by balancing only the first two terms of this equation.

We have numerically verified that the decrease of the exponent from $\tau = 2$ in the simplified model down to $\tau = 1.7$ in our original model is driven entirely by noise (unequal population sizes) resulting in a finite value of σ. A non-zero value of σ in the Eq. S5 results in $\tau < 2$. For example, if the populations at the start of each wave had a Poisson distribution so that $\sigma = \mu = 1/N$, the exponent $\tau = (1 + \sqrt{5})/2 \simeq 1.62$ would have been defined by the solution of the golden mean equation $0 = -1 + (\tau - 1) + (\tau - 1)^2$. While currently we have no first-principles argument allowing us to derive the value of σ in our basic model, the result from a Poisson distribution is not too far from the empirically observed exponent $\tau = 1.7$

Model variants

To test the robustness of our basic model with respect to rule changes we considered the following seven variants:

1. **"Neutral drift model"**.

   This variant extends our basic model by adding to our standard model the random neutral drift of population sizes (Hubbell SP (2001) The unified neutral theory of biodiversity and biogeography (MPB-32), Princeton University Press) between subsequent collapse events. To simulate this random drift, at every time step the population of each species changes up or down as prescribed by dispersion of binominal distribution: $P_i \to P_i \pm \sqrt{r \cdot P_i(1-P_i)}$, where r is the parameter quantifying the magnitude of fluctuations proportional to the inverse of the total population size and the square of the birth/death rate. After drift changes were applied to all populations we rescale them back to their carrying capacity $\sum P_i = 1$. This is followed by a collapse event as in the standard model. S1 Figure illustrates typical time courses of the diversity $D(t) = 1/\sum P_i(t)^2$ and time-aggregated species abundance distributions in this model variant for three values of r and compares them to our basic model.

2. **"Exponential fluctuations model"**, where populations are exposed to random exponential shifts between successive collapse events. In this version of the model between successive collapse events all populations exponentially shift thus adjusting the way carrying capacity is divided between the. This model is similar to the "Neutral random drift" model #1 above except that changes are proportional to P_i and not to $\sqrt{P_i}$. The population of each species is characterized by its own exponential rate $G_i(t)$ given by $n \cdot rand_i(t)$, where n quantifies the overall rate of the redistribution, while $rand_i(t)$ - a random number uniformly distributed between 0 and 1 - represents species- and time- specific shifts. We assume that differences in growth rates between species are not constant but instead fluctuate on the timescale when a single species collapses. Hence, after each collapse event we reset the exponential growth we randomly reset the rates $G_i(t)$ for all species (and not just of the collapsed one). In this version of the model we also take into account the stochastic nature of the time interval $\tau_r(t)$ between two successive collapse events, which is randomly chosen from the exponential distribution with mean value equal to 1. Thus between two successive collapse events each species population changes as $P_i \to P_i \cdot e^{G_i(t)\tau_r(t)}$ and subsequently rescaled to the carrying

capacity of the environment: $\sum P_i = 1$. As in our basic model, at every time step one randomly selected population i collapses and is reset to $P_i \to \gamma$ while all other populations are rescaled to fill up the carrying capacity: $\sum P_i = 1$. S2 Figure shows the simulations of this model for several value of n compared to our basic ($n = 0$) model.

3. **"Interconnected environments model"** with diffusion. In this version of the model there is a **single** species distributed between N local environments. As in our basic model at every time step one local population i is selected for collapse and reset to $P_i \to 0$ after which the populations are normalized back to the carrying capacity. The diffusion takes a fraction γ of the total population of 1 and distributes it equally between all local environments: $P_i \to P_i(1-\gamma) + \gamma/N$. Note, that here we implicitly assume that populations in all of these environments share the same carrying capacity. This is the case when diffusion rate of the rate-limiting nutrient is much faster then that of populations themselves. The main difference of this model from earlier variants is that populations in the lower peak with $P_i \ll 1/N$ grow approximately linearly in time (as opposed to exponentially in other versions of the model). The rate of this linear growth is the same for all species and is equal to γ/N. The exponential growth is restored for populations that are larger than average. S3 Figure shows the time-aggregated distribution of local population sizes in this model variant.

4. **"Kill-the-Winner (KtW) model"**. For bacterial populations the direction of the trend (if any) of collapse probability with population size is currently unknown. In fact one can plausible make a case for increasing of the probability of collapse with population size due to larger populations making easier to find and overall and more attractive targets for phages. In microbiology preferential targeting of large bacterial populations by virulent phages is known as "Kill-the-Winner" (KtW) hypothesis (Thingstad TF and Lignell R (1997) Aquatic Microbial Ecology 13:19-27). Here we simulate the version of our basic model where the collapse probability systematically increases with population size. At each time step we select a random population to collapse with probability $\propto P_i^\sigma$. As before the collapsing population is reset to $P_i \to \gamma$ and all populations are subsequently grown with equal exponential rates to complete saturation: $\sum P_i = 1$. S4 Figure examines time-aggregated population distributions in KtV model variant for different values of σ. Whereas small and moderate σ preserve diversity wave dynamics, the $\sigma = 1$ version does not exhibit diversity waves and predicts a population size distribution distributions, $dP/ds \propto 1/s$, or $dP/d\log(s) = constant$

(equal number of species in each decade of population sizes).

5. **"Kill-the-looser (KtL) model"** in which a collapse probability declines with population size in a power-law fashion. At each time step one select a random population to collapse with probability $\propto P_i^{-0.2}$ where P_i is the current population size of species i. In economics this corresponds to an intuitively plausible notion that larger companies are less likely to go bankrupt than smaller ones. Empirically, this trend is described by a power law with the exponent -0.2 (Nunes Amaral LA, Buldyrev SV, Havlin S, Leschhorn H, Maass P, Salinger MA, et al. (1997) Journal de Physique I. 7: 621-633.) Notice the emergence of the lower peak distribution distribution above γ that is much more narrow than in our standard model. This makes sense as in the course of each wave small populations tend to collapse over and over. These repeated collapses don't drive other populations up by much and thus their only consequence is clustering of small populations close to the very bottom of the lower peak distribution at and above γ. When the dominant upper peak population finally collapses all small populations are rescaled up to form a narrow distribution around $1/N$. This is very similar to our simplified memory-free model described in the main text and shown in Figure 4A. The new wave starts with very high diversity $D(t) \simeq N$ which is subsequently reduced with time as $D(t) = N_{surv}(t) \simeq N \exp(-t/N)$. Here we ignore a relatively small $N^{0.2}$-fold decline in collapse frequency over the range of population sizes between $1/N$ and 1. Within the same approximation each surviving population grows as $P(t) \propto \exp(t/N)$. The time-averaged distribution of populations thereby approaches the scaling regime described by:

$$\begin{aligned} \mathrm{Prob}(P_i(t) > P) &\sim \frac{1}{P} \Rightarrow \\ \frac{d\mathrm{Prob}(P_i(t) > P)}{dP} &\sim \frac{1}{P^2} \end{aligned} \tag{S6}$$

In reality the scaling exponent of the tail is around 1.8. It is the same as in our standard model but for a different reason. Indeed, taking into account that lifetime of a population before collapse scales as $1/P^{-0.2} = P^{0.2}$ one gets $\mathrm{Prob}(P_i(t) = P) \sim \frac{P^{0.2}}{P^2} = \frac{1}{P^{1.8}}$.

6. **"Fitness model"** with heterogeneous, species-specific growth rates and extinction probabilities. Each species is assigned a growth rate Ω_i used when it repopulates the freed-up carrying capacity of the environment. It also has its own extinction probability c_i. Both Ω_i and c_i are logarithmically

distributed in the interval between 0.1 and 1. That is to say their $\log_{10}$ are uniformly distributed between -1 and 0. At each time step we select one of N populations, with probability $\propto c_k$, this species goes extinct. It is immediately replaced by a new species with the population $P_k \to \gamma = 10^{-9}$, new growth rate Ω_i, and extinction probability c_i. Subsequently all of the populations $i = 1, 2...N$ are rescaled proportional to their growth rates

$$P_i \to P_i + (P_k - \gamma) \cdot \frac{\Omega_i P_i}{\sum_j \Omega_j P_j}$$

to fill up the carrying capacity of the environment $\sum P_i = 1$. The upper panel in S6 Figure shows that the time-aggregated population distribution in this model preserves its power-law tail, whereas lower panel illustrates that in order for a species to reach substantial population size its fitness parameters need to be particularly favorable. Indeed, populations larger than $1/N = 0.001$ tend to have smaller than average extinction probabilities c_i, and larger than average growth rates Ω_i.

7. **"Resilience model"** where heterogeneous, species-specific growth rates and survival ratios (population reduction following a collapse) are competing with each other. Each species is assigned a growth rate $\Omega_i \in [0.1, 1]$ and collapse size $\gamma_i \in [10^{-9}, 10^{-2}]$, both logarithmically distributed (uniform distribution of the logarithm of the variable). At each time step we select one of N populations, and collapse its population $P_k \to \gamma_k \cdot P_k$. Note that unlike in previous versions we scale down the population proportional to its size and not proportional to the carrying capacity of the environment. This reflects a different interpretation away from our basic model, where a collapse represents not an extinction of the species followed by the appearance of a new species at a fixed (very small) population size. In the new version of the model a collapse represents a sudden but proportionate reduction of a population e.g. due to species' phenotypic or genotypic bet-hedging. In this version of the model an extinction happens only if a very low population is reached, i.e. when $\gamma_k P_k < 10^{-9}$. If this lower bound is reached the old species goes extinct and a new species with the initial population $P_k = 10^{-9}$ is introduced. The new species is assigned new random values of Ω_k and γ_k. As in the previous model following each collapse all populations $i = 1, 2...N$ are rescaled proportional to their growth rates $P_i \to P_i + (P_k - \max(\gamma_k P_k, 10^{-9})) \cdot \frac{\Omega_i P_i}{\sum_j \Omega_j P_j}$ to fill up the carrying capacity of the environment: $\sum P_i = 1$.
