# Supplementary figures and images for: Diversity Waves in Collapse-Driven Population Dynamics
Source: PLoS Comput Biol. 2015 Sep 14;11(9):e1004440. doi: 10.1371/journal.pcbi.1004440 (PMC4569562; doi:10.1371/journal.pcbi.1004440)

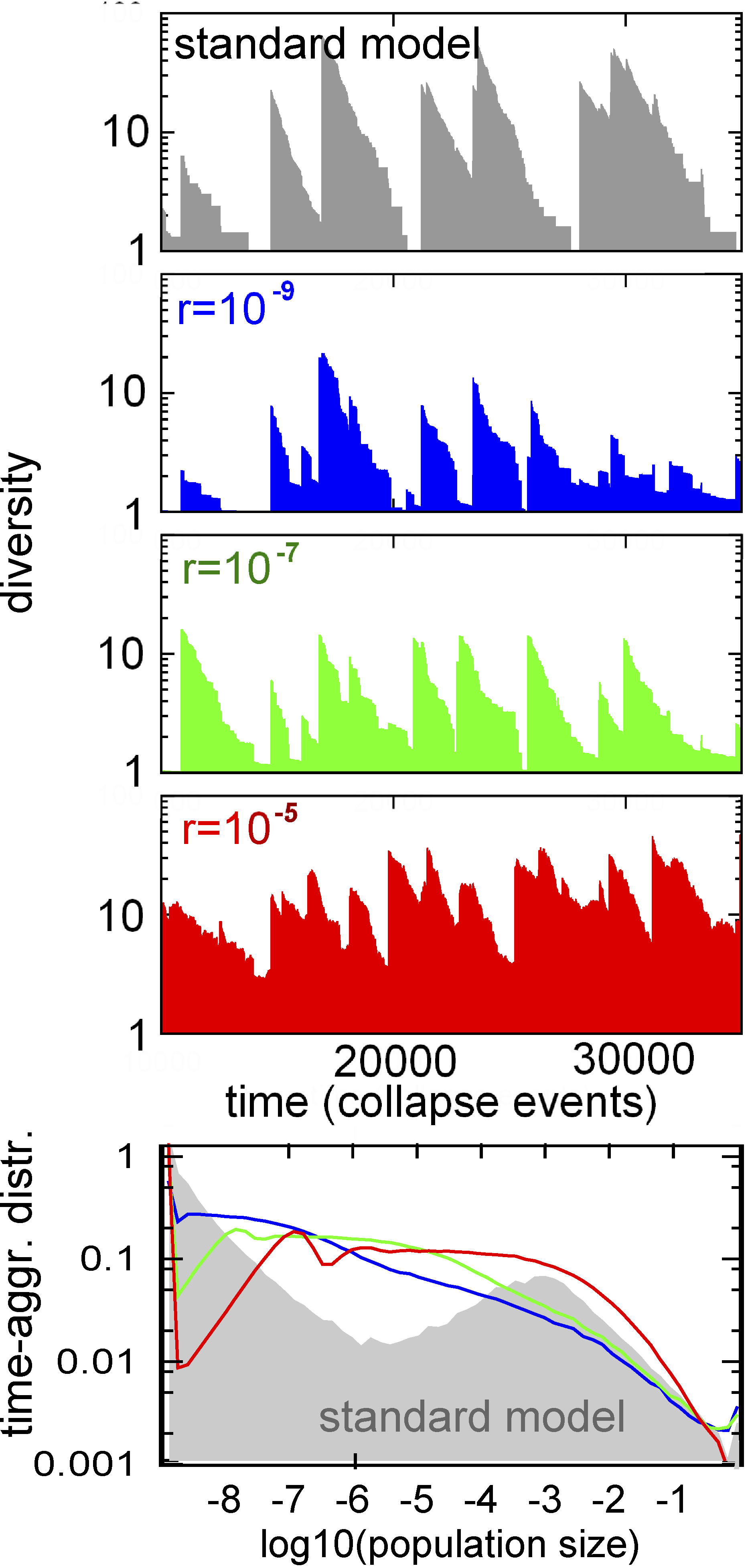

Supplement: S1 Fig — This variant extends our basic model with N = 1000 and collapse ratio γ = 10−9 by adding the neutral drift at rate r taking place between subsequent collapse events in our standard model: Pi→Pi±r⋅Pi(1−Pi). The lower panel shows the time-aggregated distributions in our system simulated for 106 collapse events. The grey shaded area refers to our basic, unmodified model, i.e. to the r = 0 case, while three color lines correspond to r = 10−9 (blue), r = 10−7 (green), and r = 10−5 (red). The upper four panels illustrate typical time courses of the diversity D(t) = 1/∑P i(t)2 in our basic model and for three values of the rate r color-coded as in the lower panel. (TIFF) [file pcbi.1004440.s002.tiff]

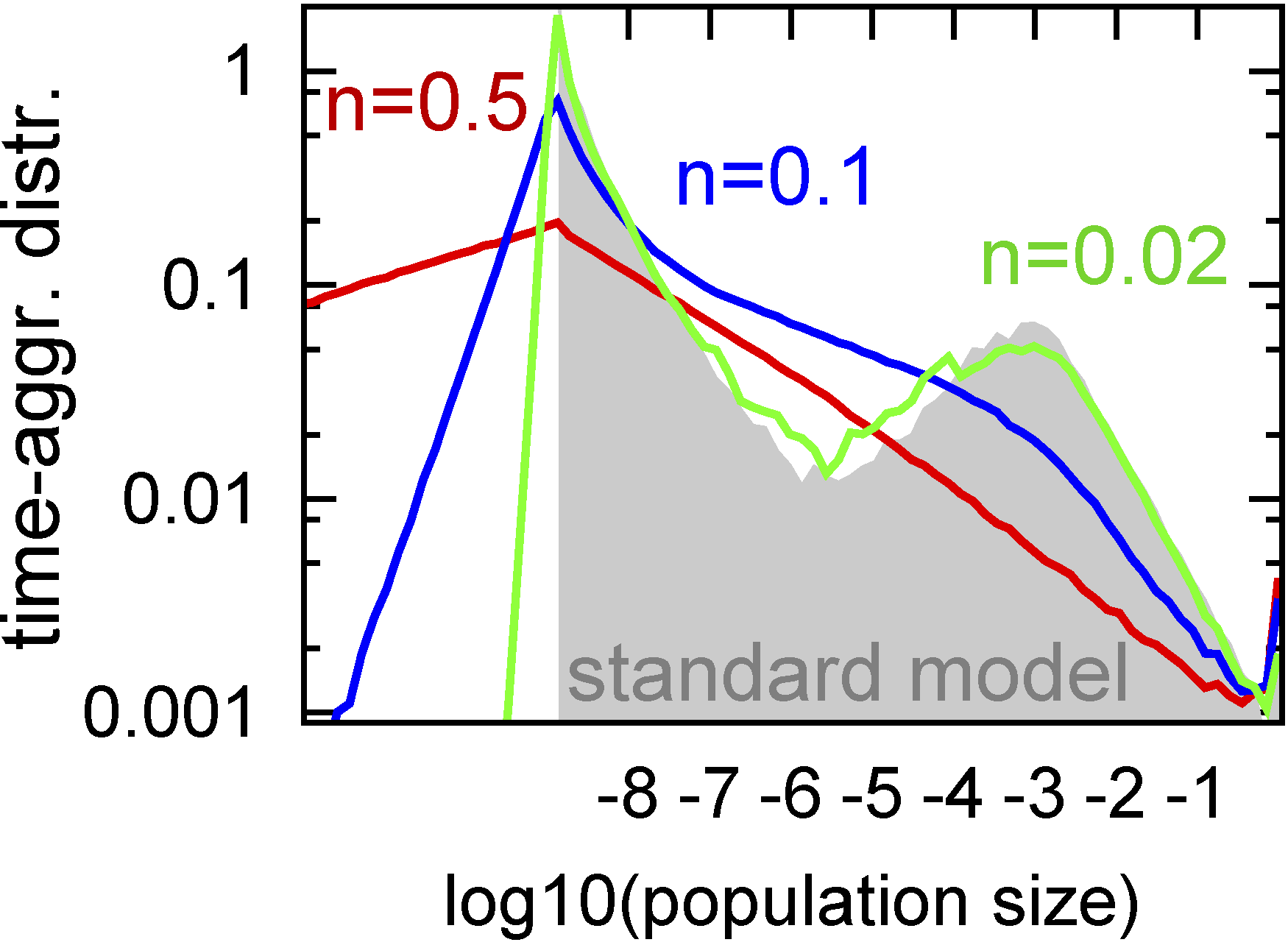

Supplement: S2 Fig — Figure shows this model with N = 1000, γ = 10−9, and n = 0.02 (green), n = 0.1 (blue), n = 0.5 (red) system simulated for 106 collapse events. The grey shaded area shows the time-aggregated population distribution in our basic model, corresponding to the n = 0 limit. (TIFF) [file pcbi.1004440.s003.tiff]

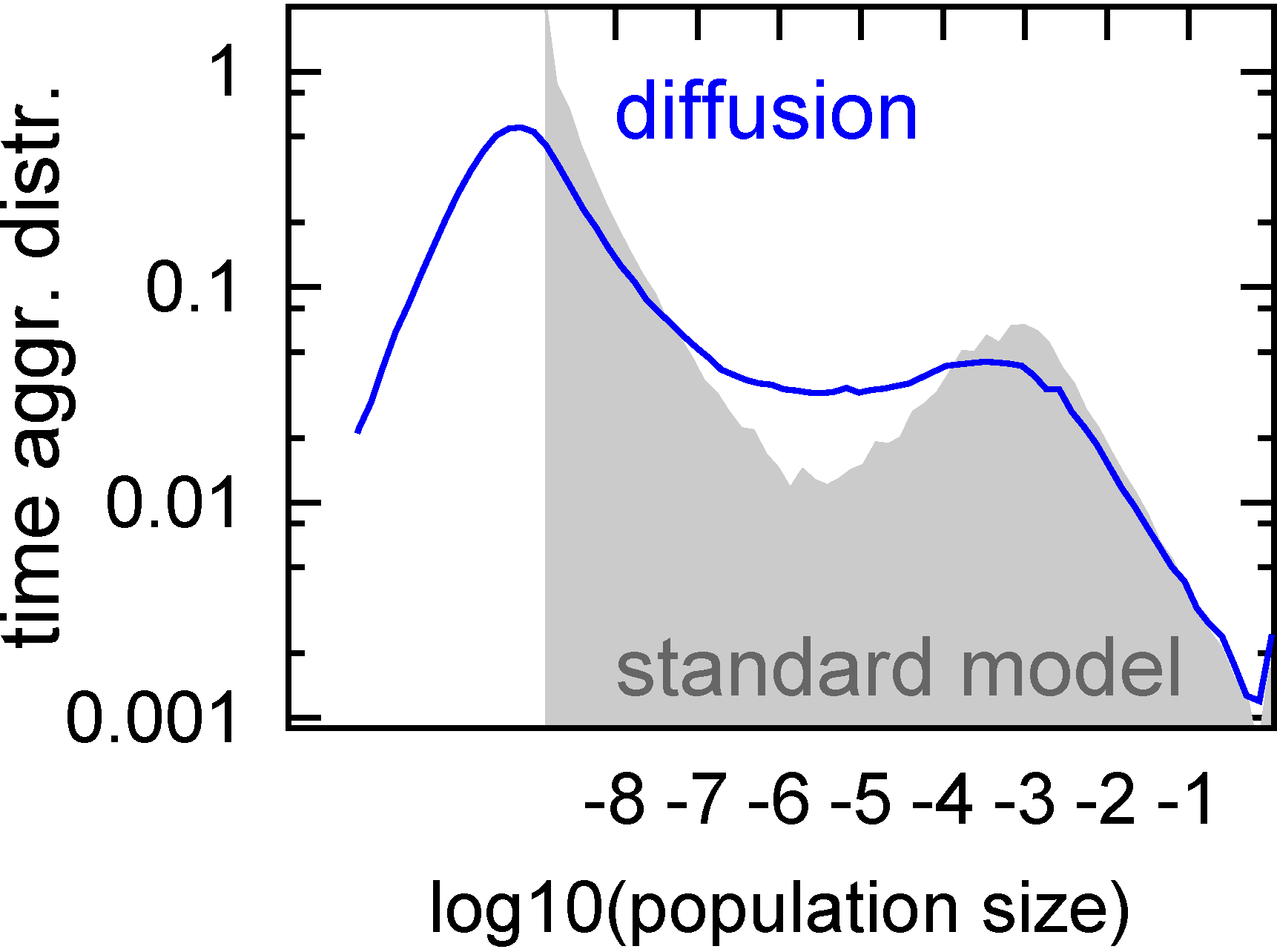

Supplement: S3 Fig — Figure shows time-aggregated species abundance distributions in the model with N = 1000 environments connected by diffusion of strength γ = 10−9 simulated over 106 collapse events. The basic model with the same parameters is shown as the grey shaded area. (TIFF) [file pcbi.1004440.s004.tiff]

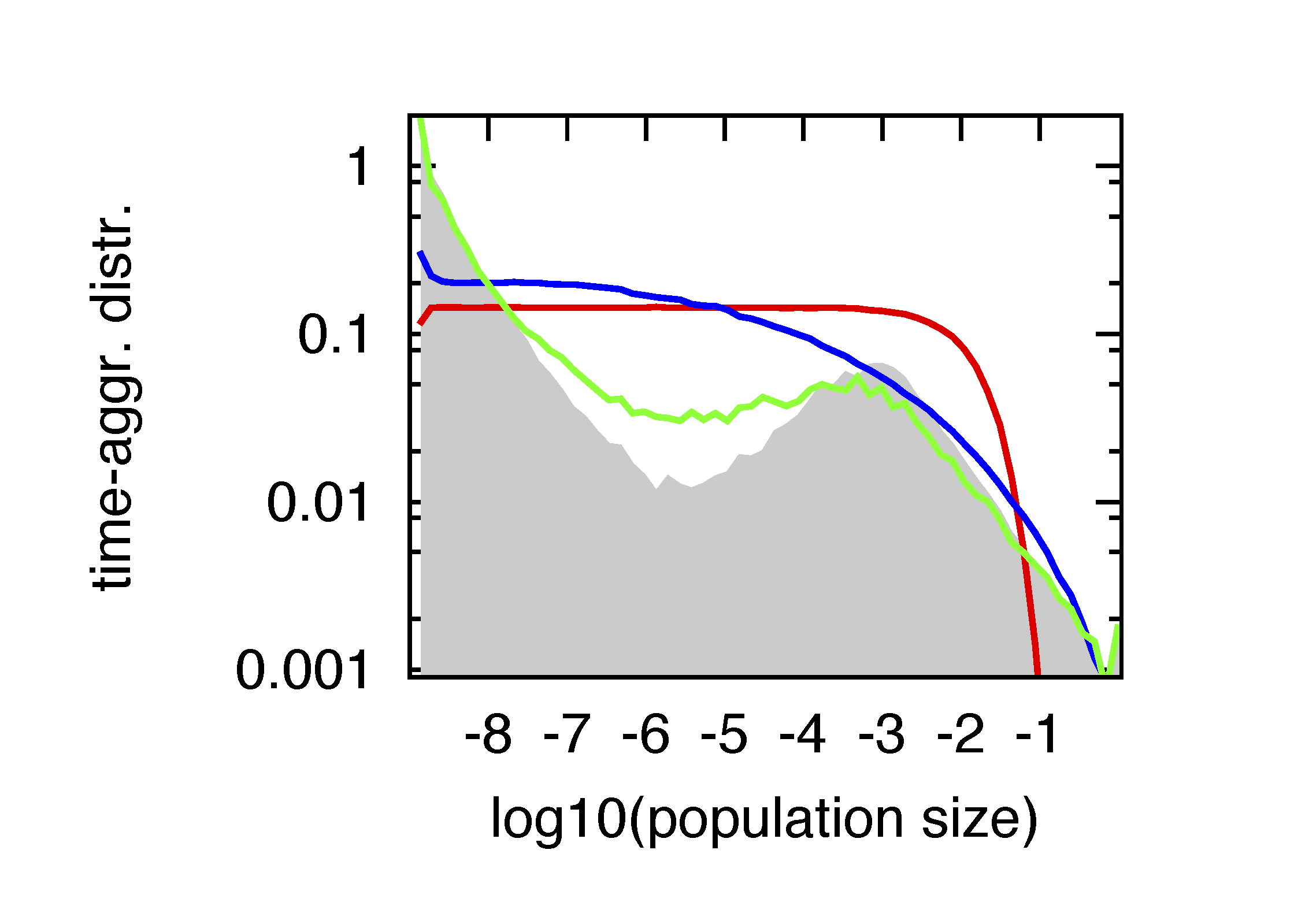

Supplement: S4 Fig — Model variant in which larger populations are preferentially targeted for collapse: ci∝Piσ. Different colors correspond to time-aggregated SADs in the model with N = 1000, γ = 10−9, and σ = 0.01 (green), 0.2 (blue), and σ = 1.0 (red) simulated over 5 ⋅ 106 collapse events. The grey shaded area refers to time-aggregated population distribution in our basic, unmodified model with the same N and γ. (TIFF) [file pcbi.1004440.s005.tiff]

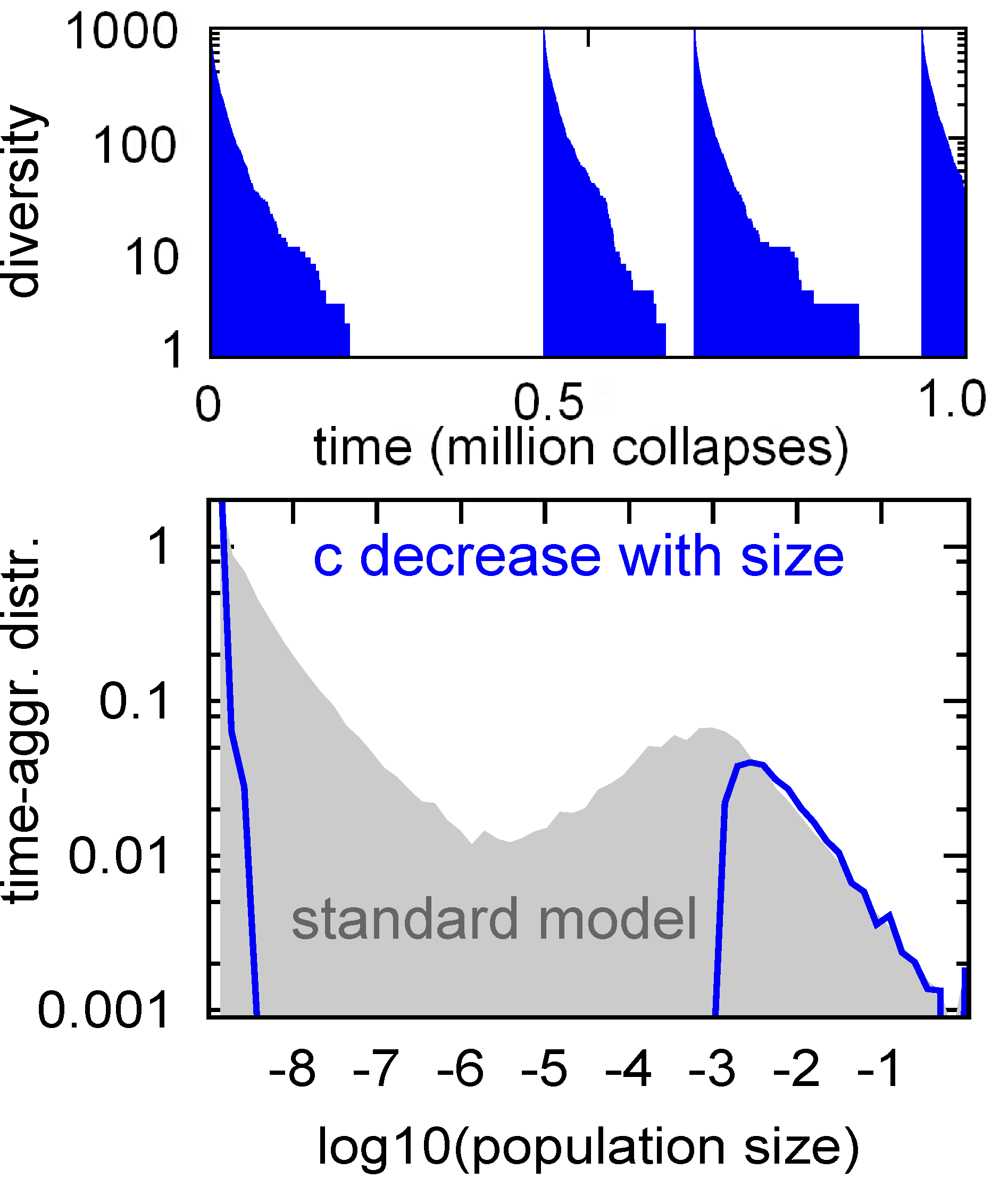

Supplement: S5 Fig — Model variant in which the collapse probability declines with population size as a power law with exponent -0.2. The figure shows an N = 1000, γ = 10−9 system simulated for 106 collapse events. The upper panel illustrates the recurrent diversity waves, whereas the lower panel shows time-aggregated distributions, with the grey shaded area referring to our standard model. (TIFF) [file pcbi.1004440.s006.tiff]

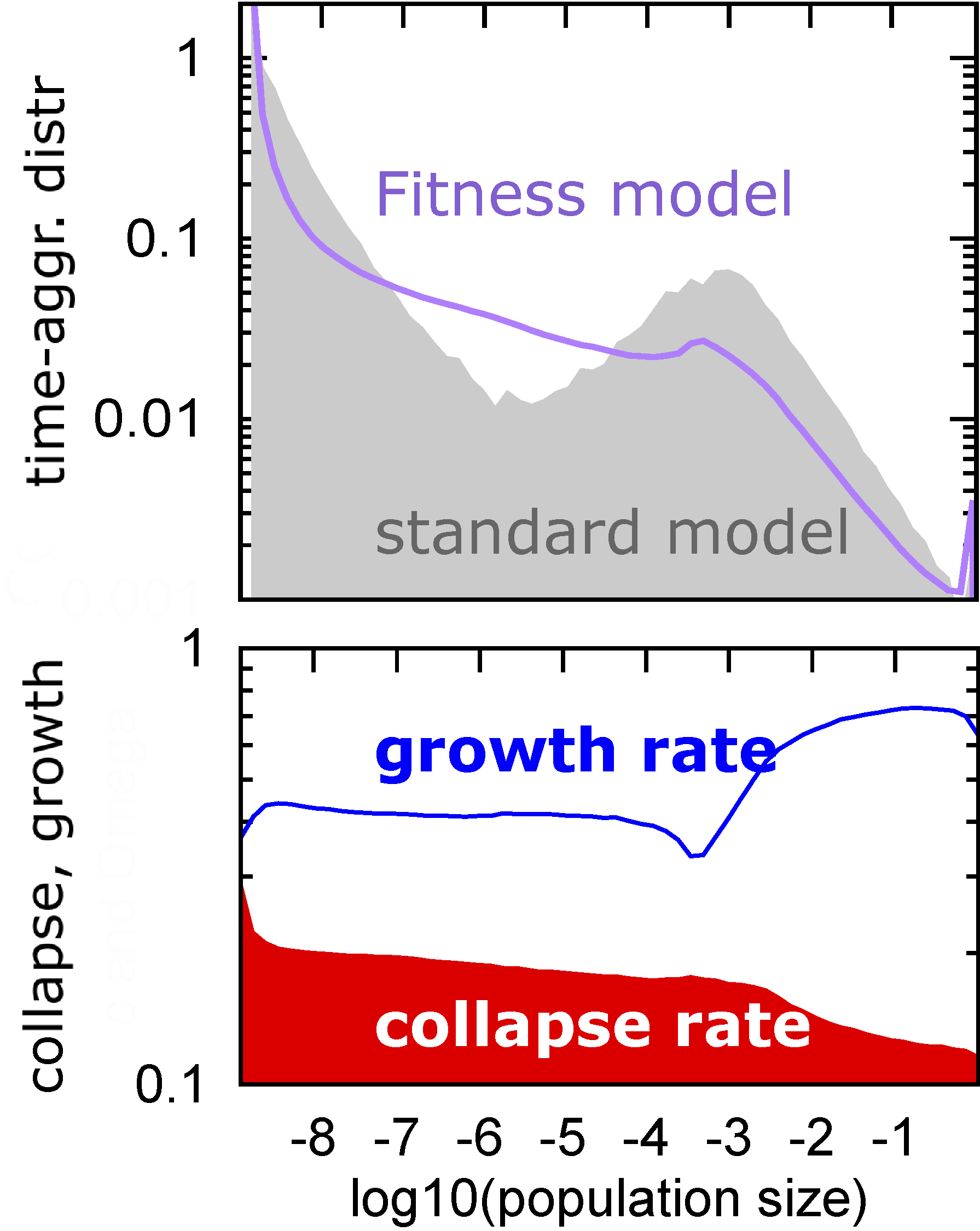

Supplement: S6 Fig — Model variant with heterogeneous, species-specific growth rates and extinction probabilities. Each species is assigned a growth rate Ωi used when it repopulates the freed-up carrying capacity of the environment. It also has its own collapse probability c i. Both Ωi and c i are logarithmically distributed in the interval between 0.1 and 1. The purple curve in the upper panel shows the time-aggregated population distribution whereas the grey shaded area is that for the standard model where species’ growth and collapse rates are all equal to each other. The lower panel shows the average growth rate ⟨Ωi⟩ (blue) and the average collapse probability ⟨c i⟩ (red shaded area) of species binned by their collected at every time step. Both curves represent time-aggregated averages of individual populations. (TIFF) [file pcbi.1004440.s007.tiff]

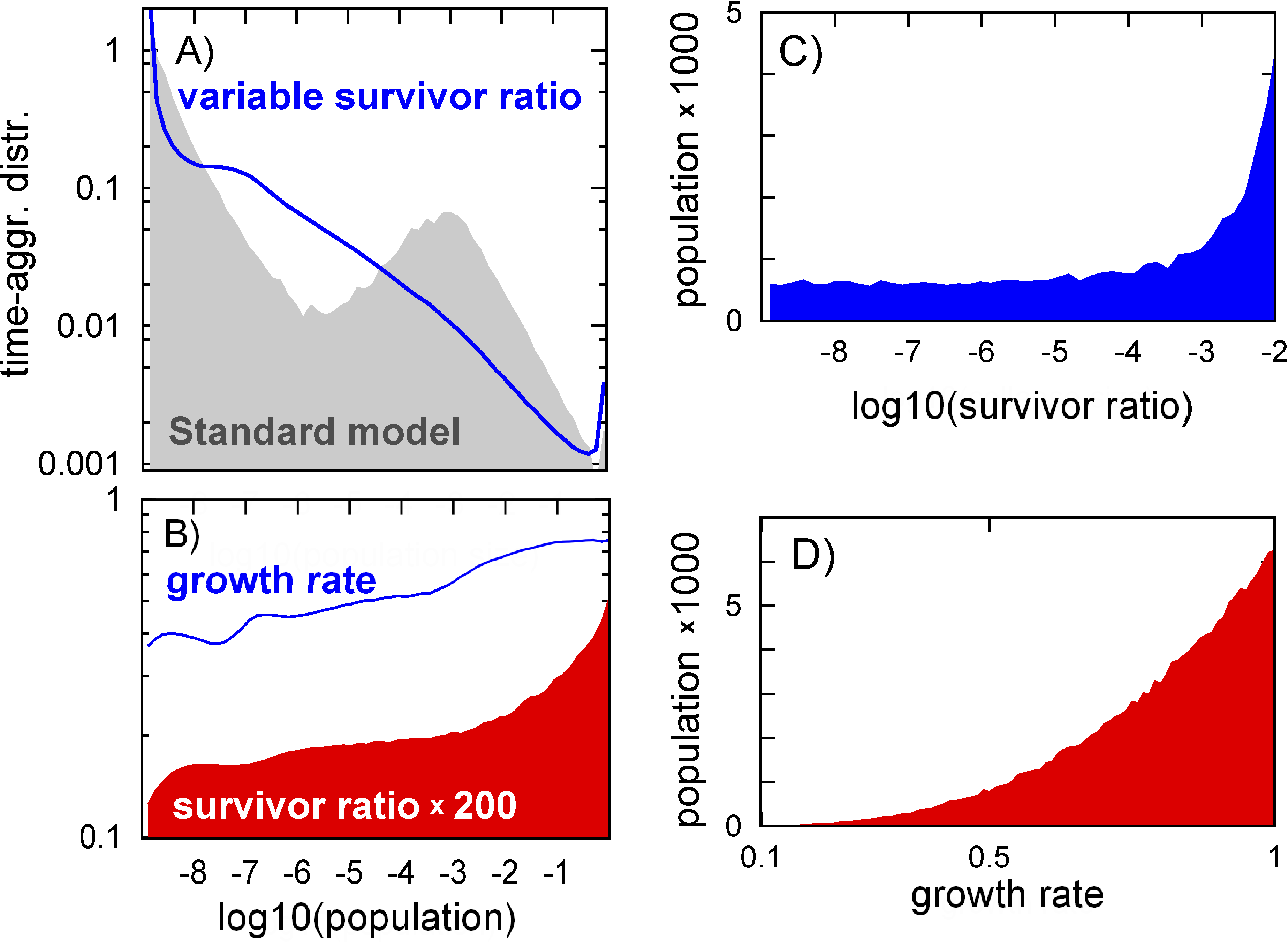

Supplement: S7 Fig — Model variant with heterogeneous, species-specific growth rates and survival ratios following a collapse. Each of N = 1000 species is assigned a growth rate Ωi ∈ [0.1,1] and collapse size γ i ∈ [10−9,10−2], both logarithmically distributed. A) The blue curve shows the time-aggregated population distribution, whereas the grey area refers to that in our basic model. B) The average growth rate ⟨Ωi⟩ (blue) and the average survival ratio ⟨γ i⟩ multiplied by 200 (red shaded area) binned by the population size collected at every time step. Both curves represent time-aggregated averages of individual populations. C) The average (arithmetic) population size as a function of species’ survivor ratio γ i. D) The average (arithmetic) population size as a function of species’ growth rate Ωi. (TIFF) [file pcbi.1004440.s008.tiff]
